# Global research trends on epigenetics and cancer: A bibliometric analysis

**DOI:** 10.1097/MD.0000000000043197

**Published:** 2025-08-15

**Authors:** Xinyi Zhong, Du Chen

**Affiliations:** a Department of Basic Medical Sciences, Medical College, Shaoguan University, Shaoguan, China.

**Keywords:** bibliometrics, cancer, citespace, epigenetic, VOSviewer

## Abstract

**Background:**

Epigenetics in cancer has been a focus of intense research in the recent years. This phenomenon has attracted great interest because it offers potential targets for cancer therapy. To capture the intellectual landscape of this field, this article conducted a bibliometric analysis to assess the current status, frontiers and future trends of epigenetic research in cancers.

**Methods:**

A bibliometric analysis was performed using data from the Web of Science Core Collection up to 2023. Analytical tools such as CiteSpace, VOSviewer, and the R package “bibliometrix” were employed for comprehensive data analysis and visualization. This process identified the publication of the articles, countries, authors, institutions, journals and keywords.

**Results:**

The results showed that there were 51,742 articles from the WoSCC database during 1985 to 2023 for cancer epigenetics. The number of epigenetic cancer-related publications has increased annually. The most contributed country is the United States, with 850,726 citations and 55 average article citations. China ranks second, with 413,386 citations and an average of 44.7. The most productive institutions were from the USA. *Plos One* (1020), *International Journal of Molecular Sciences* (957), and *Cancers* (945) were the top 3 contributing journals. The top 3 most common keywords were “DNA methylation,” “expression” and “cancer.” Research hotspots within these themes highlight intensive areas of study and the potential for significant breakthroughs.

**Conclusions:**

This study presents an in-depth overview of the current state of epigenetics in cancers research. And the purpose of this review will be to understand the characteristics of bibliometric analyses of epigenetic cancers and anticipate the progress in understanding this field.

## 1. Introduction

Cancer is a major global social, public health and economic problem, causing huge social and economic losses. There were close nearly 20 million new cancer cases and 9.7 million deaths from cancer in the year 2022, and according to the prediction, the number of new cancer cases worldwide may reach 35 million or more by 2050.^[1]^ The exact mechanisms underlying all of the cancers still is a mysterious world. Moreover, due to the diverse and atypical clinical manifestations of cancer, the diagnosis, treatment and prognosis are affected. Therefore, it is very important to find the pathogenesis of cancer to prevent and treat cancer.

“Epigenetics” is a concept proposed by developmental biologist Conrad H. Waddington (1905–1975) in 1942, referring to the complex of developmental processes between genotype and phenotype.^[2]^ Epigenetic regulation, which involves reversible and heritable changes in gene-expression that occur in response to external stimuli without altering the DNA sequence, is fundamental to understanding the pathogenesis of various diseases, including cancer.^[3,4]^ This regulation includes DNA methylation, chromatin remodeling, histone modifications, and the expression of diverse noncoding RNAs (ncRNAs), such as microRNAs (miRNAs), long ncRNAs (lncRNAs), and circular RNAs (circRNAs).^[5,6]^ Although the exact mechanism behind cancer is still unknown, it is believed that there are genetic factors involved.^[7]^ And there are many studies have found that epigenetics is essential for elucidating the intricate mechanisms underlying the development and progression of cancer.^[4]^

The growing interest in the role of epigenetics in cancer is evident from the rising number of publications. However, a comprehensive bibliometric analysis of this body of work is lacking. Bibliometric analysis is an information visualization method to identify and summarize the frontiers or hot spots in a certain area.^[8]^ Moreover, the researcher can compare the research status among various institutions, authors, or journals, evaluate the latest cutting-edge research, understand the scientific articles, and visualize their trends through this method.

This study presents a detailed bibliometric analysis of scientific literature on epigenetics in cancer from 1985 to 2023, utilizing data from the Web of Science Core Collection (WOSCC). The research aims to show the developments and future trends in the research field of epigenetics in cancer and offer valuable insights for future investigations.

## 2. Materials and methods

### 2.1. Data source

**Figure 1.**
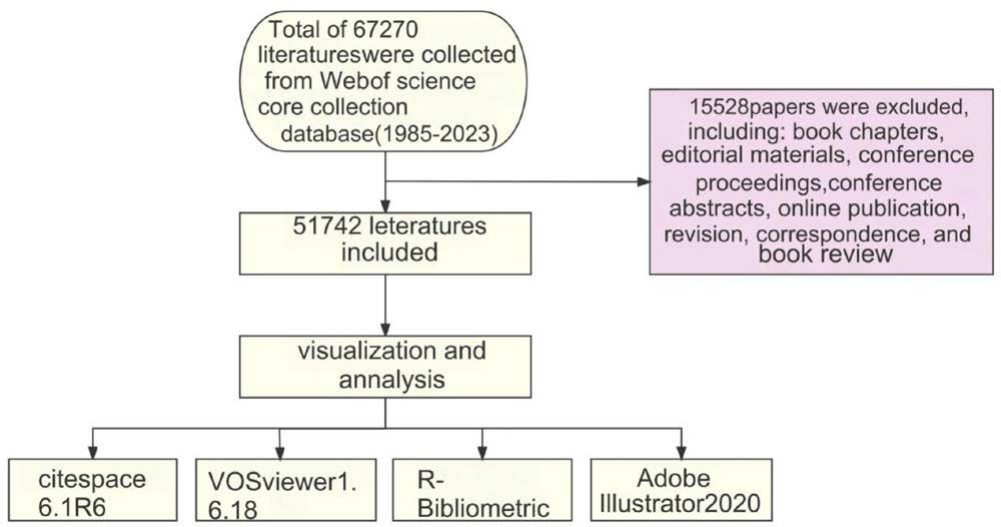
Flow diagram for searches of databases.

Search for the data in the WoSCC, which is regarded as one of the most widely used and comprehensive databases for citation analysis. A single researcher performed the search independently, restricting the publication date to the period between January 1, 1985, and December 31, 2023. The keywords for searching the articles were: TS = (“epigenetic”) AND TS = (“cancer” or “tumor” or “malignancy” or “neoplasm” or “sarcoma” or “carcinoma” or “tumor”). The search was performed on October 28, 2023, and 67,270 articles were obtained in total. The following were excluded from the final selection: book chapters, editorial materials, conference papers, conference abstracts, online publications, revisions, letters, book reviews, aiming to exclude unsuitable topics and research purposes. The language was not limited. Thus, a total of 51,742 papers and reviews were ultimately selected. The data was exported in plain text format and named as “download.” See Figure 1 for details.

### 2.2. Visualization analysis

For the visualization analysis of the data, CiteSpace (version 6.1.6R), VOSviewer (version 1.6.18), R-Bibliometric and Adobe Illustrator2020 are utilized.^[9]^ CiteSpace, VOSviewer and Adobe Illustrator are used to demonstrate cluster analysis, burst detection, timeline analysis, and journal co-occurrence analysis of references. VOSviewer (1.6.11) was used to create institutions network visualizations, co-authorship, co-citation authors, and keywords and explore collaboration networks between authors/institutes/countries/journals.^[10]^ In VOSviewer, nodes were used to represent countries, institutions, journals, and authors, and their size was determined by their co-occurrence frequency in titles and abstracts.^[11]^ Besides, Adobe Illustrator software was used to generate geographical distribution maps of publication outputs based on year and country, among others.

## 3. Results

### 3.1. Overall analysis of publications and citations

**Figure 2.**
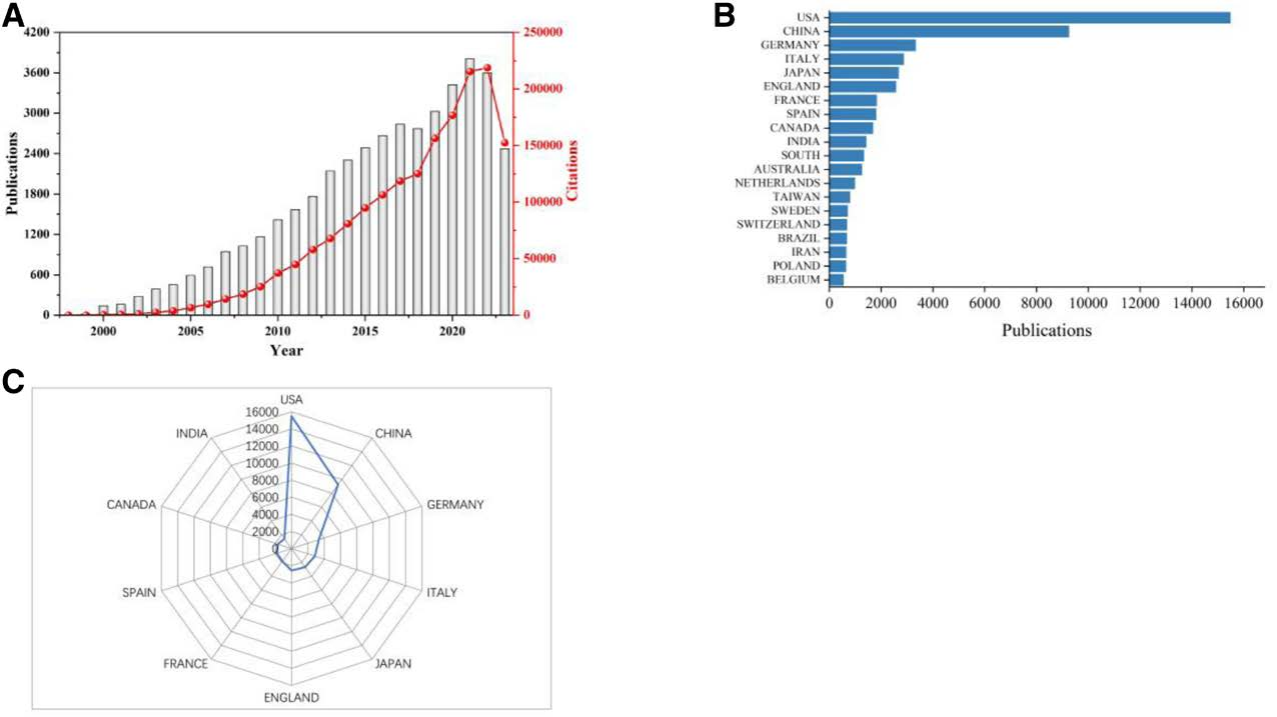
(A) Trend of publications and citations of epigenetic research in cancers from 1998 to 2023. (B) Bar graph of the top 20 productive countries. (C) Total publications radar map of the top 10 countries.

The number of research papers published in different periods demonstrates the popularity and development tendency of research in a particular field. There were 51,742 articles from the WoSCC database during 1985 to 2023 for cancer epigenetics. The quantity of publications, citation ranks and average citation ranks per year are shown in Table 1. And it also shows the mean total number of citations (TC) per article. Table 1 shows that articles had the highest average citation ranks were published in 2023, with 61.62. The peak of average citation rank per year is 2021, with 8973. The quantitative analysis of published articles about epigenetics in cancers is shown in Figure 2. An upward trend in annual publications is observed in Figure 2A, reaching a peak in 2021 before a slight decline, yet maintaining high overall numbers. Furthermore, the quantity of different countries/regions articles were statistically analyzed. The total publication ranks of the top 20 countries/regions is displayed in Figure 2B. And Figure 2C presents total publication ranks from the 10 leading countries/regions. The result shows that the United States leads with 15,479 publications (29.92%), followed by China (9248 publications, 17.87%). These results reflect that cancer-related epigenetic research has attracted significant attention and scholarly recognition.

**Table 1:** Global publication output and citation trend of epigenetic in cancers.

Year	Mean TC per article	Publications	Mean TC per year	Total citations
1998	7.38	16	118	118
1999	47.43	7	166	332
2000	3.78	138	174	521
2001	4.62	162	187	748
2002	4.05	279	226	1130
2003	6.60	385	424	2541
2004	8.42	453	545	3814
2005	11.21	591	828	6623
2006	13.48	718	1076	9681
2007	15.15	943	1429	14,288
2008	18.17	1027	1696	18,656
2009	21.72	1162	2103	25,238
2010	26.27	1416	2861	37,199
2011	28.63	1568	3207	44,898
2012	32.98	1763	3876	58,141
2013	31.66	2141	4237	67,792
2014	35.12	2303	4758	80,892
2015	38.12	2487	5266	94,794
2016	39.87	2667	5597	106,336
2017	41.82	2836	5930	118,591
2018	45.13	2771	5955	125,051
2019	51.63	3027	7104	156,282
2020	51.66	3422	7686	176,774
2021	56.58	3806	8973	215,347
2022	60.80	3598	8751	218,765
2023	61.62	2473	5861	152,393

TC = total number of citations.

### 3.2. Contributions of countries/regions

**Figure 3.**
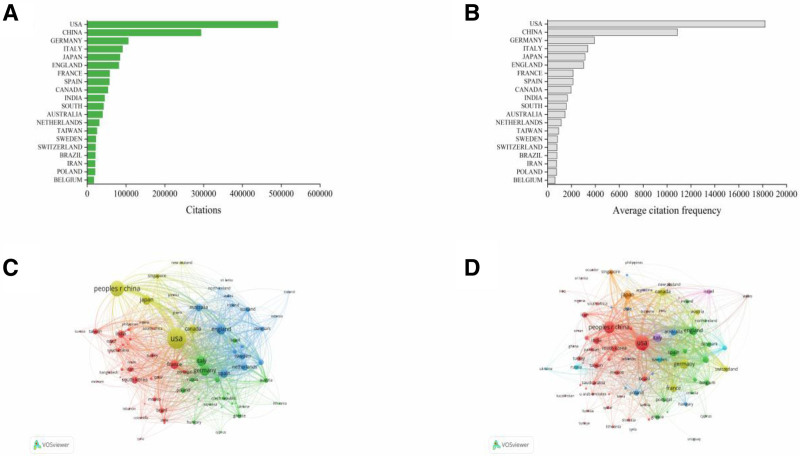
(A) Map of cumulative citations across the 20 leading countries from 1985 to 2023. (B) The country-region-specific citation average numbers from 1985 to 2023. (C) The countries/regions cooperation networks visualization map generated by using VOS viewer. (D) The countries/regions citation network visualization map generated by using VOSviewer.

During the period from 1985 to 2023, 450 countries and regions published research publications related to epigenetic cancers. Table 2 presents the 10 countries with the highest citation counts. Similarly, Figure 3A and B shows the total and average number of citations for the top 20 countries, respectively. The most contributed country is the United States, with 850,726 citations and 55 average article citations. China ranks second, with 413,386 citations and an average of 44.7. The international cooperation analysis is shown in Figure 3C. The United States has the most cooperation with many countries, China ranks second. However, the level of cooperation among other countries remains low relatively. The research analyzed the network of global collaborative with a minimum threshold of 450 papers by using VOSviewer. As shown in Figure 3D, the largest total link strength (TLS) of the United States ranks first (TLS = 136,525), followed by China (TLS = 63,904) and Germany (TLS = 23,005). In addition, the United States having the highest proportion of international cooperation (0.45), followed by China and Germany.

**Table 2:** Productive countries/regions related to epigenetic in cancers.

Country	Articles	SCP	MCP	MCP-ratio	Citations	Average article citations	TLS
USA	15,479	8505	6974	0.45	850,726	55	136,525
China	9248	5166	4082	0.44	413,386	44.7	63,904
Germany	3334	2179	1155	0.35	141,528	42.5	23,005
Italy	2866	2062	804	0.28	125,961	44	24,676
Japan	2667	1852	815	0.31	91,745	34.4	19,869
England	2569	1809	760	0.3	104,918	40.8	22,376
France	1824	1361	463	0.25	60,356	33.1	15,540
Spain	1806	1290	516	0.29	72,999	40.4	16,236
Canada	1684	1146	538	0.32	88,191	52.4	10,424
India	1417	984	433	0.31	43,148	30.5	12,059

MCP = number of publications co-authored by authors from different countries, SCP = number of publications co-authored by authors from the same country, TLS = total link strength.

### 3.3. Analysis of main publishing institutions and authors

There were 450 institutions participated in research on epigenetic in cancers. The most productive institutions were from the USA. As shown in Table 3, among the top 10 productive institutions, 7 come from the USA, 2 are from France and 1 from Germany. Harvard University (2055) was the most productive institution, followed by the University of California system (1934) and the University of Texas system (1818).

**Table 3:** The top 10 most productive institutions related to epigenetic in cancers.

Affiliation	Articles	Country	TLS
Harvard University	2055	USA	30,825
University of California System	1934	USA	40,614
University of Texas System	1818	USA	25,452
Udice French Research Universities	1575	France	17,325
National Institutes of Health NIH USA	1560	USA	23,400
Institut National de la Sante et de la Recherche Medicale Inserm	1356	France	28,476
Harvard Medical School	1344	USA	20,160
Helmholtz Association	1237	Germany	24,740
Johns Hopkins University	1168	USA	15,184
Utmd Anderson Cancer Center	1145	USA	16,030

TLS = total link strength.

**Figure 4.**
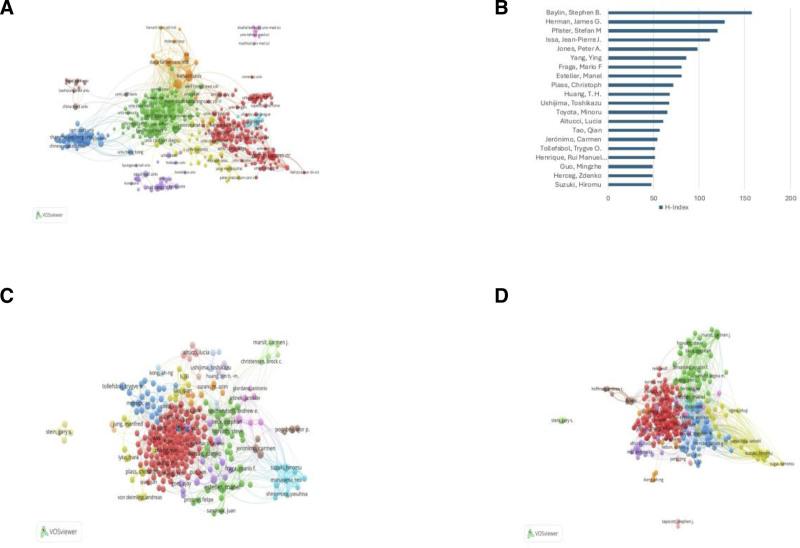
(A) Collaborative visualization network of institutions constructed using VOSviewer software. (B) Map of the leading 20 authors with the highest H-index. (C) The visualization network of author cooperation. (D) Visualization map of co-cited authors generated by VOSviewer.

Additionally, the research used VOS viewer to analyze the cooperation among institutions. Harvard University has the strongest collaboration capabilities, as shown in Figure 4A. The world’s top universities and institutions have made outstanding contributions to the development of the field.

In total, 352 authors and 221,835 co-cited authors were identified. Tables 4 and 5 summarize the top 10 most generative authors and the top 10 co-cited authors, respectively. Esteller, Manel, Herman, James G and Baylin, Stephen B ranked in the top 3 with 264, 141 and 122 papers respectively. While Yang, Ying, Pfister, Stefan M and Jones, Peter A. published fewer papers, their total citations were as high as 63,402, 62,089 and 49,740, respectively. The H-index is a measure of a scholar’s overall influence, representing the number of publications cited at least N times (N). Table 4A and Figure 4B show Baylin, Stephen B has the highest H index, which is 158, which having great influence in this field. In addition, an author’s contribution to a particular field is presented by the “article classification,” representing the author’s publications in that field as a percentage of all publications. The 2 biggest contributors in the field were Esteller, Manel and Herman, James G., with 62.26 and 33.98 respectively. Figure 4C is a visualization map of author co-authorship analysis generated by VOSviewer.

**Table 4:** The top 10 most productive authors related to epigenetic in cancers.

Author	Publications	H-index	G-index	M-index	Citations	Articles fractionalized
Esteller, Manel	264	81	207	13.45	28,576	62.26
Herman, James G.	141	128	193	10.58	92,294	33.98
Baylin, Stephen B.	122	158	178	9.97	149,455	32.53
Issa, Jean-Pierre J.	120	112	158	8.62	51,659	28.99
Huang, T. H.	101	68	139	8.19	15,147	28.45
Fraga, Mario F	95	81	136	8.06	28,576	23.00
Jerónimo, Carmen	94	54	124	7.26	10,579	21.61
Henrique, Rui Manuel Ferreira	83	52	118	6.74	10,283	22.02
Tao, Qian	79	57	110	6.04	8750	20.68
Yang, Ying	79	86	103	5.77	63,402	18.59

**Table 5:** The top 10 most productive co-cite author related to epigenetics in cancers.

Co-cite author	Citation	TLS	Centrality
Esteller, Manel	6346	24,950	0.18
Baylin, Stephen B.	4433	22,894	0.15
Henrique, Rui Manuel Ferreira	1574	20,440	0.12
Suzuki, Hiromu	2949	18,039	0.09
Jones, Peter A.	4466	17,980	0.09
Herceg, Zdenko	3167	17,853	0.09
Jerónimo, Carmen	1019	14,486	0.06
Herman, James G.	310	14,164	0.04
Issa, Jean-Pierre J.	902	13,509	0.03
Fraga, Mario F	4818	12,758	0.03

TLS = total link strength.

The top author with the highest number of citations is Baylin, Stephen B, with 149,455. In addition, Figure 4D shows the co-cited author network visualized using VOSviewer, and the highest TLS author is Esteller, Manel (TLS = 24959), followed by Baylin, Stephen B (TLS = 22,894) and Henrique, Rui Manerl Ferreira.

### 3.4. Analysis of the top publishing journals

**Figure 5.**
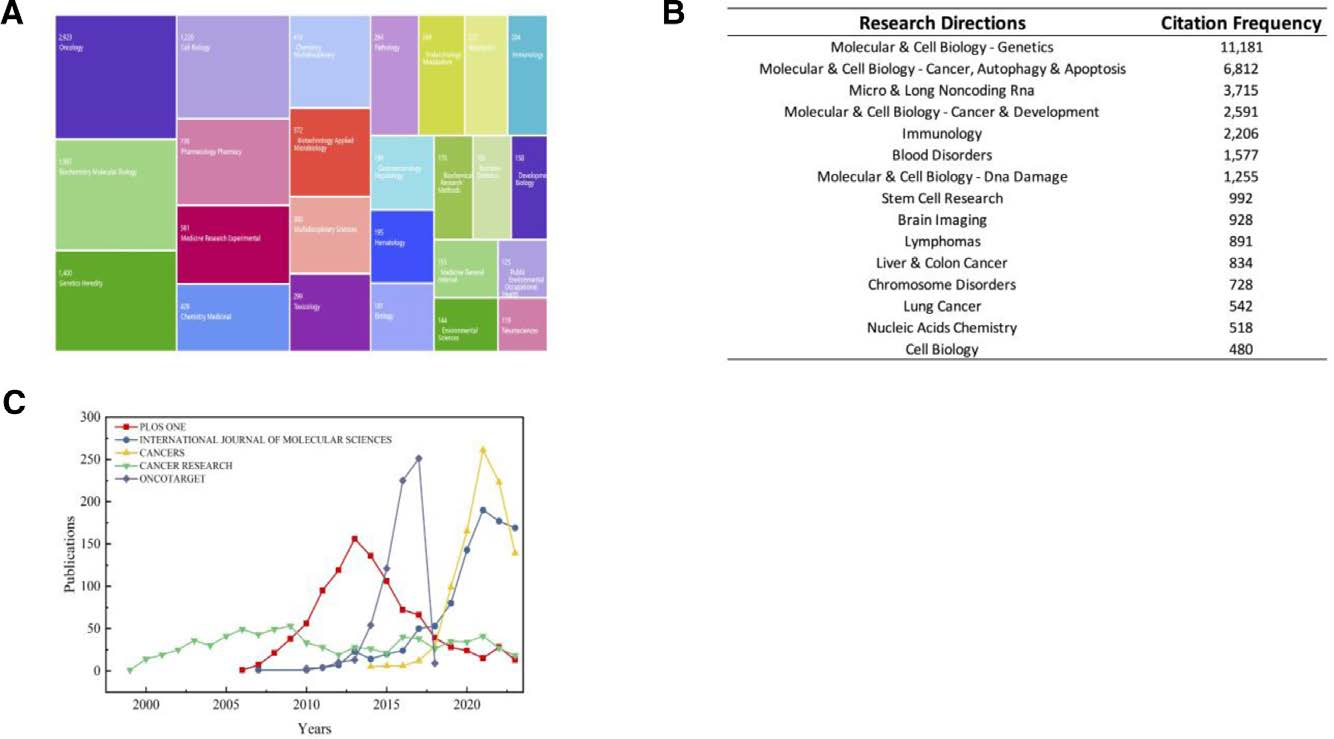
(A) The top 25 most dominant directions of the research in this study. (B) The top 15 most cited research directions in this study. (C) The chart of the leading 5 journals with the most publications in terms of annual publications changes from 1998 to 2023.

Highly cited papers are those that have been referenced frequently by other publications, which are pivotal contributions within the academic literature that have significantly influenced the landscape of a particular field. The top 10 publishing journals are shown in Table 6. *Plos One* (1020), *International Journal of Molecular Sciences* (957), and *Cancers* (945) were the top 3 contributing journals. Among the top 10 journals, their impact factors were relatively high, with 60% being higher than 5.0. Besides this, *Plos One* had both the highest citations and the highest number of H-index. R software was used to conduct the top 25 most popular research directions visualization analysis, as Figure 5A performed. Top 15 citation of research directions are shown in Figure 5B. Molecular & Cell Biology-Genetics are the top research directions in this field. Figure 5C shows the publication trends annually of the top 5 most generative journals. *Cancers* exhibits the highest growth rate, *Oncotarget* ranks second, while they all show a downward trend in recent years. Besides, Table 7 presents the top 10 most cited articles with the realm of epigenetics in cancers. The highest citation count of article titled “Molecular origins of cancer: Epigenetics in cancer” by Esteller et al has (2710) and was released in the *New England Journal of Medicine* (IF = 158.5). The article published in *Boinformatics* (IF = 5.8), titled “Minfi: a flexible and comprehensive Bioconductor package for the analysis of Infinium DNA methylation microarrays” ranks second, which has been cited 2389 times.

**Table 6:** Top 10 journals related to the research of epigenetics in cancers.

Rank	Journal	Publications	H-index	Total citations	IF (2023)
1	PLoS One	1020	64	34,336	3.700
2	International Journal of Molecular Sciences	957	31	32,782	6.208
3	Cancers	945	56	32,042	6.575
4	Cancer Research	774	54	28,120	13.312
5	Oncotarget	690	49	24,753	6.000
6	Oncogene	688	47	24,568	4.419
7	Scientific Reports	632	43	18,500	4.600
8	Nature Communications	625	41	18,426	16.600
9	Epigenetics	575	26	18,204	2.149
10	Clinical Epigenetics	560	24	17,649	7.259

IF = impact factor.

**Table 7:** Top 10 articles with the most citations concerning the research of Epigenetic in cancers.

Paper	Journal	Citations	Corresponding author	IF (2023)
Molecular origins of cancer: Epigenetics in cancer	New England Journal of Medicine	2710	Esteller, M	158.5
Minfi: a flexible and comprehensive Bioconductor package for the analysis of Infinium DNA methylation microarrays	Boinformatics	2389	Aryee, MJ	5.8
Cancer Epigenetics: From Mechanism to Therapy	Cell	2300	Dawson, MA	64.5
Epigenetic modifications and human disease	Nature Biotechnology	2298	Portela, A	46.9
Selective Inhibition of Tumor Oncogenes by Disruption of Super-Enhancers	Cell	2101	Lovén, J	64.5
Genome-wide Methylation Profiles Reveal Quantitative Views of Human Aging Rates	Molecular Cell	1914	Hannum, G	16.0
Epigenetics in cancer	Carcinogenesis	1911	Sharma, S	4.7
Environmental epigenomics and disease susceptibility	Nature Reviews Genetics	1904	Jirtle, RL	42.7
PD-L1 (B7-H1) and PD-1 pathway blockade for cancer therapy: Mechanisms, response biomarkers, and combinations	Science Translational Medicine	1771	Zou, WP	17.1
Cancer epigenomics: DNA methylomes and histone-modification maps	Nature Reviews Genetics	1702	Esteller, M	42.7

IF = impact factor.

### 3.5. Reference analysis

**Figure 6.**
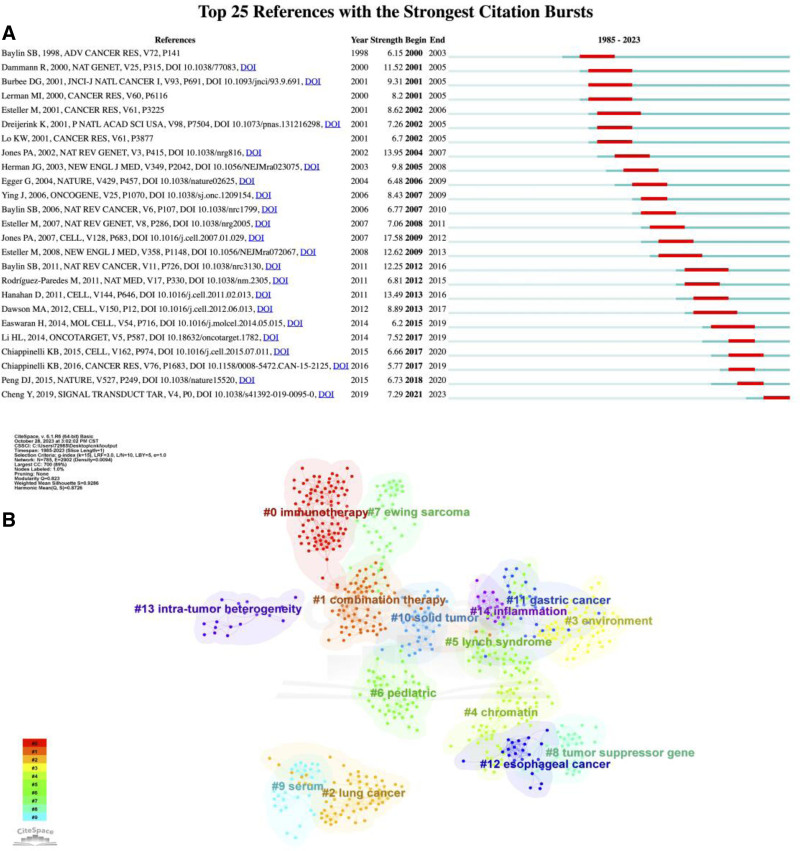
(A) Top 25 references with the strongest citation bursts. (B) Evolution path map of major themes generated by CiteSpace software.

The study cited a total of 116,521 studies related to cancer epigenetics. The leading 25 strongest Citation bursts references are summarized in Figure 6A. The reference citation burst in this study began in 2000 due to the paper published by Baylin SB in 1998. The latest reference citation burst was detected in 2021 and last until now. Among them, a review article entitled “The Epigenomics of Cancer” by Jones PA published in *Cell* had the intensity with highest burst in 2007, attaining 17.58. Currently, most important articles are still cited frequently and it can be speculated that epigenetic of cancers research will still be a research hotspot in the next few years. Furthermore, the co-cited collaboration network is shown in Figure 6B, which reveals a diverse network of topics in cancer research, covering therapeutic strategy innovation (immunotherapy, combination therapy), environmental and inflammatory drivers, tumor heterogeneity challenges, and the focus on specific cancer types (pediatric, gastric cancer), providing researchers with a panoramic view of the field’s development.

### 3.6. Analysis of keywords and subject terms

**Figure 7.**
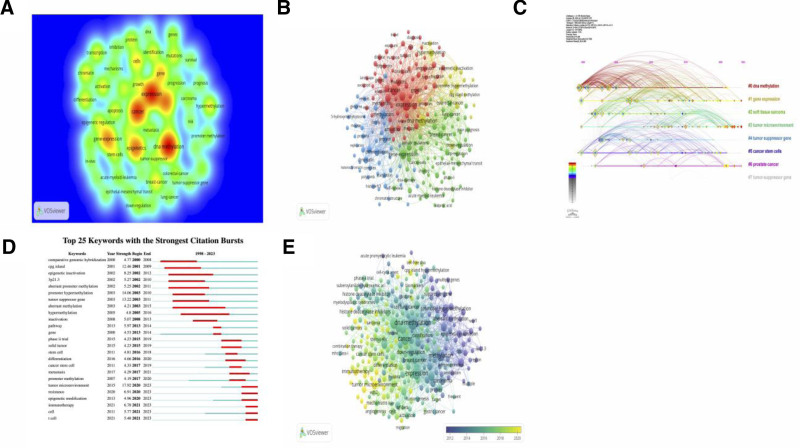
(A) The top 50 keywords with the highest frequency. (B) Keyword cluster map constructed via VOSviewer. (C) Temporal evolution of keywords was analyzed through CiteSpace software. (D) Top 25 keywords with the strongest citation burst. (E) The trend of subject evolution from 1998 to 2023.

According to the keyword analysis, future research directions and hotspots can be predicted to support scientific research decision-making. A total of 85,855 keywords were included in this study. Table 8 summarizes the top 20 keywords with the highest frequency. The top 3 most common keywords were “DNA methylation,” “expression” and “cancer.” Figure 7A shows the heat map of the 50 keywords with the highest occurrence rates. Figure 7B illustrates the categorization of keywords into 4 distinct color-coded clusters. DNA methylation, expression, and cancer are among the frequently used terms. Figure 7C shows the top 8 frequency keyword timeline perspective. “DNA methylation” exhibits the highest magnitude and statistically significant sustained visibility. The current hot spots are “gene expression,” “tumor microenvironment” and “prostate cancer.” Figure 7D presents the top 25 keywords with the strongest citation bursts. “Comparative genomic hybridization,” the earliest keyword burst, was detected in 2000. Later, researches related to genome and epigenetic mechanism such as “CpG island” or “promoter hypermethylation” became hot topics. The strength of “tumor microenvironment” hit 17.92 (2020–2023), which indicates the research in this field has increased rapidly in recent years. Recent popular topics such as “resistance” (2020–2023), “epigenetic modification” (2020–2023), “immunotherapy” (2021–2023), “cell” (2021–2023) and “t cell” (2021–2023) have bursts that extend until 2023, suggesting that these research topics might become new research foci in the next few years. Figure 7E shows the trend of subject evolution from 1998 to 2023. “Histone deacetylase inhibitors,” “methylation” evolved into “immunotherapy,” “cell-free DNA,” “tumor microenvironment.” To sum up, Figure 7 shows the analysis of the keywords, which depicts the evolution of research interests over time, and reflects research frontiers, hot topics, emerging trends.

**Table 8:** Top 20 keywords with the most frequencies concerning the research of epigenetics in cancers.

Rank	Keywords	Frequency	TLS	Rank	Keywords	Frequency	TLS
1	DNA methylation	12,146	31,628	11	Hypermethylation	2583	8758
2	Expression	11,414	31,127	12	Differentiation	2530	6679
3	Cancer	9964	25,611	13	Activation	2477	7070
4	Epigenetics	6762	18,308	14	Identification	2460	7048
5	Methylation	6652	18,845	15	Transcription	2406	6475
6	Gene-expression	5781	12,995	16	Apoptosis	2373	7084
7	Gene	4083	11,932	17	Mutations	2359	5852
8	Cells	3192	9081	18	Epigenetic regulation	2283	5766
9	Breast-cancer	2781	7525	19	Protein	2216	5969
10	Chromatin	2648	6597	20	Growth	2122	6594

TLS = total link strength.

## 4. Discussion

### 4.1. General information discussion

This article conducted a bibliometric analysis to assess the current status, the frontier hotspots and trends of epigenetic research in cancers. The results of this study showed that the number of publications each year has been steadily increasing (Fig. 2). This reflects the continued interest. Although the circulation in 2023 decreased slightly, it still exceeded 2000, which shows the ongoing interest in cancer epigenetics. The United States, China and Germany rank in the top 3 in terms of publication and citations. The United States ranks first in the number of papers published, cited frequency and average cited frequency, and dominates the field. Because of its very strong economic strength and beneficial policy and scientific support, the United States is the largest country in this field of research. At the same time, although China is a developing country, it has an important position in the field of epigenetics research.

According to the results of countries/regions distribution, the most contributed country is the United States, with 850,726 citations and 55 average article citations (Fig. 3). And the United States has the most cooperation with many countries, China ranks second. However, the level of cooperation among other countries remains low relatively. The United States leads in cross-national impact metrics and also the highest MCP-Ratio (Table 2), which indicates that American researchers work closely with researchers in other countries.

According to research institutions, 7 of the top 10 most published institutions are from the United States (Table 3), which interprets why the United States makes contributions to a huge number of epigenetic and cancer-related articles. In addition, France accounts for 20 percent of the top 10 universities. Unfortunately, there is no any single Chinese university in the top 10.

In author and co-cited author analysis, Esteller, Manel, Herman, James G and Baylin, Stephen B ranked in the top 3 with 264, 141 and 122 papers respectively. While Yang, Ying, Pfister, Stefan M and Jones, Peter A. published fewer papers, their total citations were as high as 63,402, 62,089 and 49,740, respectively. From the visualization map of co-cited author, Esteller, Manel has the highest TLS (Fig. 4). More important articles may be published by the above team members, strengthening cooperation with these top teams is a good choice for research.

Identification of important journals analysis, *Plos One*, *International Journal of Molecular Sciences*, *Cancers*, *Cancer Research* and *Oncotarget* are probably the main publishing places for cancer-related articles (Table 6). It is recommended to submit more cancer-related articles to these journals. Among the most prolific publications, there are 2 journals had an IF of more than 10: *Cancer Research* (IF 2023, 13.312) and *Nature Communications* (IF 2023, 16.6). *Cancers* exhibits the highest growth rate, *Oncotarget* ranks second, while they all show a downward trend in recent years. Of particular note is that are it is an amazing choice for researchers to publish epigenetics and cancer research in these top 10 journals in the future. The most cited references are often considered the basis of research in a particular field. The most cited article is “Molecular Origins of Cancer: The Epigenetics of Cancer” (Table 7). This example illustrates how to improve cancer diagnosis and treatment from a molecular perspective.

### 4.2. Hotspots and frontiers of epigenetic in cancers

Bibliometrics analysis can reflect the hot spots and frontiers of this research field. Through a multiple analysis of references and keywords, these results showed that molecular and cell biology-genetics is the most popular research direction in epigenetics of cancer (Fig. 5). Through the citation analysis of reference, the result showed that the most of the topics focus on the relationship between cancer, epigenetics, etiology, diagnosis, and treatment (Table 7). According to the keyword analysis, the top 3 most common keywords were “DNA methylation,” “expression” and “cancer” (Table 8). “DNA methylation” has the largest and consistently attracting high attention (Fig. 7C). “Comparative genomic hybridization,” the earliest keyword burst, was detected in 2000. Later, researches related to genome and epigenetic mechanism such as “CpG island” or “promoter hypermethylation” became hot topics (Fig. 7D). As DNA-methylation research advances, it shows the increasingly importance of epigenetics in DNA-methylation function and cancer development.^[12,13]^ Multiple studies have found that the inactivation of certain tumor suppressor genes is the result of hypermethylation of promoter regions, and that DNA methylation leads to cancer by causing widespread gene silencing.^[14,15]^ In addition, DNA methylation is one of the most intensively studied epigenetic modifications in mammals, which fully demonstrates that it is a current research hotspot.^[16]^ For example, histone modifications have been shown to be closely related to DNA methylation, regulating genome function by altering chromatin structure.^[17]^ Global hypomethylation, which induces genomic instability, also contributes to cell transformation. Noncoding RNAs, such as microRNAs, have been found to play a key role in DNA methylation.^[18]^ Covalent addition of methyl groups usually occurs in cytosine within CpG dinucleotides, which are concentrated in large clusters called CpG islands. It suggests that CpG can be used as a potential target for cancer treatment.^[19]^ In conclusion, a comprehensive grasp of the epigenetic mechanism of cancer affords potential targets for the engineering of new treatments. Ancer is characterized by abnormal expression in many aspects. Through analysis, the study has identified several keywords with the strongest citation bursts. “Comparative genomic hybridization,” the earliest keyword burst, was detected in 2000. Later, researches related to genome and epigenetic mechanism such as “CPG island” or “promoter hypermethylation” became hot topics. One of the recent popular topics is “cell” (2021–2023) (Fig. 7D), which plays a very important role in the occurrence and development of cancer. For example, stem cells, often defined as cloned cells capable of self-renewal and multi-lineage differentiation, are biological tissue units responsible for the development and regeneration of tissue and organ systems. There is evidence that tissue-specific stem cells are the cells of origin for many types of cancer.^[20,21]^ For example, cell subpopulations with CD34- and CD38- phenotypes similar to normal hematopoietic stem cells (HSCs) were found to be able to initiate human acute myeloid leukemia suggesting that normal HSCs are targets for leukemia transformation.^[22,23]^ In addition, a number of mutations in leukemia cells were found in normal HSCs suggesting that stem cells are common targets for leukemia transformation.^[24]^ In addition, several studies have shown that under standard culture conditions, postnatal stem cells can transform into malignant cells without any genetic manipulation.^[25]^ For example, mesenchymal stem cells enter a malignant transformation state after long-term in vitro culture and form tumors in vivo. Taken together, abnormal epigenetics are closely linked to the pathogenesis of cancer, some of which have shown promise as markers of diagnosis and disease activity. The best-studied epigenetic changes in cancer are the methylation changes that occur within the CpG island, and the next generation sequencing platform has now provided a genome-wide map of CpG methylation.^[26]^ And, recent cancer genome sequencing has identified recurrent mutations in DNA methyltransferase 3A (DNMT3A) in up to 25% of patients with acute myeloid leukemia. Moreover, Ten-eleven-translocation-2 (TET2) mutations also confer enhanced self-renewal properties on malignant clones. Histone acetylation: n ε-acetylation of lysine residues is a major histone modification involved in transcription, chromatin structure, and DNA repair. Acetylation neutralizes the positive charge of lysine and thus may weaken the electrostatic interaction between histones and negatively charged DNA. Enzymes that modify histones have been found, such as Lysine acetyltransferases (KATs), the first to be shown.^[27]^ Taken together, current research focuses on DNA methylation, cancer, epigenetics, methylation, gene-expression, gene, cells, etc, through which researchers try to explore the pathogenesis of cancer.

### 4.3. Research frontiers and future trends of epigenetics in cancer

Bibliometrics analysis can reflect research frontiers and future trends of this research field. Based on multiple analyses of keywords and subject terms, the top 2 frequently cited keywords are “DNA methylation” and “expression,” showing that it remains a key focus of cancer and epigenetics research (Fig. 7A). Abnormal gene-expression regulation is a key element in tumorigenesis and development, and epigenetic mechanism are influential in the regulation of gene-expression.^[28,29]^ In the genetic susceptibility to cancer, maternal inherited mitochondrial DNA (mtDNA) is believed to be involved in cancer development and prognosis, and is considered to be a therapeutic target for cancer treatment.^[30]^ This suggests that researchers should focus more on exploring epigenetic networks and the specific role of epigenetic inheritance in different types of cancer in the future, and provide a theoretical basis for developing personalized treatment strategies.

However, the hot spot evolved from “DNA methylation” to “gene expression” and “tumor microenvironment” (Fig. 7C). DNA methylation regulates gene-expression by recruiting proteins involved in gene suppression or inhibiting the binding of transcription factors to DNA. At present, there is compelling evidence that long noncoding RNAs (lncRNAs) mediate DNA methylation under both physiological and pathological conditions. Moreover, DNA-methylation canyons are preferentially hypermethylated in cancer. Most of the DNA-methylation canyons are labeled by H3K27me3 and are associated with Polycomb proteins such as EED, EZH2, RING1B. DNA methylation in cancer cells shifts the pattern of gene-expression inhibition from a plastic polycomb-dependent H3K27Me3-mediated pattern to a more stable DNA-methylation-dependent pattern of events. Therefore, the control of DNA hypermethylation of certain oncogenes and the control of DNA damage-dependent methylation of CpG islands can help to mitigate the development of cancer. Moreover, DNA-methylation inhibitors have become a mainstay in the treatment of some hematological malignancies.^[31]^ In addition to their ability to reactivate genes that acquire DNA methylation during cancer development, including tumor suppressors, they induce the expression of thousands of transposable elements, including endogenous retroviruses and latent cancer testicular antigens that are normally silenced by DNA methylation in most somatic cells.

From the visualization map of top 25 keywords with the strongest citation bursts in epigenetic research of cancers, the strength of “tumor microenvironment” hit 17.92 (2020–2023) (Fig. 7D), which indicates the research in this field has increased rapidly in recent years. The trend of subject evolution from 1998 to 2023 showed that “histone deacetylase inhibitors,” “methylation” evolved into “immunotherapy,” “cell-free DNA,” “tumor microenvironment” (Fig. 7E). The tumor microenvironment not only contains cancer cells and already altered cellular structures, but is also capable to recruit normal cells and release cytokines to sustain the function of tumor microenvironment. Tumor microenvironment targets the local immune response in solid tumors to help immune cells entering the tumor and function efficiently within the tumor.^[32]^ Modulation of the tumor microenvironment is of special significance in cancer immunotherapy. Epigenetic alterations are known contributors to cancer development and aggressiveness. Additional to alterations in cancer cells, aberrant epigenetic marks are present in cells of the tumor microenvironment, including lymphocytes and tumor-associated macrophages.^[33,34]^ Tumor microenvironment is of great interest because of the potential roles it plays in supporting or moderating tumor growth. In certain cancers with extensive stroma (e.g., PDAC and breast-cancer), stroma-targeted therapies are being considered and tested.^[35–37]^

And recent popular topics such as “resistance” (2020–2023), “epigenetic modification” (2020–2023), “immunotherapy” (2021–2023), “cell” (2021–2023) and “t cell” (2021–2023) have bursts that extend until 2023 (Fig. 7D), suggesting that these research topics might become new research foci in the next few years. Epigenetic modifications involve changes in chromatin structure, the methylation status of DNA fragments, and chemical transformations of histone chromosomal proteins (acetylation, methylation, ADP-ribosylation, ubiquitination, and phosphorylation), and the regulation of noncoding RNAs (ncRNAs). Several studies have demonstrated that epigenetic modifications are one of the main mechanisms underlying many diseases, including cancer. These diseases experience significant deregulation of all epigenetic components, which both affect protein expression regulation and the modification of their functions. This deregulation determines the epigenetic signature of cancer.^[38]^ Modulating epigenetics in combination with immunotherapy might be a promising therapeutic option to improve the success of this therapy. Further studies are necessary to understand in depth the impact of the epigenetic machinery in the tumor microenvironment.

In summary, aberrant gene function and altered patterns of gene-expression are key features of cancer. And growing evidence shows that epigenetic abnormalities participate with genetic alterations to cause this dysregulation. The latest research focus on DNA methylation, epigenetic modification, tumor microenvironment and so on. The in-depth research in these fields is expected to reveal new mechanisms and provide new breakthroughs in the diagnosis and treatment of cancer.

## 5. Limitations

There were some limitations in this study. First, only publications from WoSCC were included in this study, which may have excluded some valuable information. Second, important non-English papers may be overlooked, leading to research bias and decreased credibility. Third, recently published articles have not had enough time to be cited.

## 6. Conclusion

Our bibliometric analysis provides a comprehensive overview of the current state of research in cancer epigenetics, identifying key themes, hotspots, and directions for future research. It will offer suggestions for researchers in selecting new research directions, such as epigenetic regulation and DNA methylation. This timely review scrutinizes research trends and hotspots on epigenetic cancers, which could progress the field and form the basis for forthcoming studies.

## Acknowledgments

We would like to thank Web of Science Core Collection for providing the raw data for this study.

## Author contributions

**Data curation:** Xinyi Zhong, Du Chen.

**Funding acquisition:** Du Chen.

**Visualization:** Xinyi Zhong.

**Writing – original draft:** Xinyi Zhong.

**Writing – review & editing:** Du Chen.
